# Comparative Epidemiological and Metabolic Profiling in Alcoholic Versus Metabolic Dysfunction-Associated Steatotic Liver Disease: A Western India Cross-Sectional Analysis

**DOI:** 10.7759/cureus.111938

**Published:** 2026-07-02

**Authors:** Sushant S Dhanavade, Mandakini S Kshirsagar, Axita C Vani

**Affiliations:** 1 Biochemistry, Krishna Institute of Medical Sciences, Krishna Vishwa Vidyapeeth (Deemed to be University), Karad, IND

**Keywords:** alcoholic liver disease, comorbidity, epidemiology, india, masld, metabolic syndrome

## Abstract

Introduction: The global burden of steatotic liver disease is escalating, with alcoholic liver disease (ALD) and metabolic dysfunction‑associated steatotic liver disease (MASLD) representing distinct etiological pathways. Recent nomenclature changes and evolving epidemiology necessitate contemporary comparative analyses, particularly in understudied populations such as India. This study aimed to characterize and compare the demographic, anthropometric, and metabolic profiles of patients with alcoholic fatty liver disease (AFLD) and MASLD in Western India.

Methods: A hospital‑based analytical cross‑sectional study was conducted from June 2023 to December 2024. Participants were stratified into three groups: AFLD (n=30), MASLD (n=30), and healthy controls (n=30). Sex distribution was comparable across groups, but age was not formally matched; instead, age was included as a covariate in regression analyses to adjust for potential confounding. Diagnosis was ultrasonographically confirmed. Comprehensive clinical data, including metabolic comorbidities, were analyzed using analysis of variance (ANOVA), chi‑square tests, and binary logistic regression (SPSS v25.0, released 2017; IBM Corp., Armonk, New York, United States, α=0.05).

Results: Significant inter‑group differences emerged: Patients with MASLD presented younger (45.1±9.8 years) versus AFLD (52.5±10.2 years, p=0.032). Both groups exhibited obesity (body mass index (BMI): AFLD 31.2±3.5 kg/m², MASLD 32.8±4.1 kg/m², p<0.001 versus controls 24.5±2.8 kg/m²). Metabolic comorbidities clustered differently: diabetes prevalence was 46.7% (14/30) in MASLD versus 40.0% (12/30) in AFLD (p=0.002 versus controls). Hypertension affected 53.3% (16/30) of patients with MASLD and 50.0% (15/30) of patients with AFLD (p=0.005). Dyslipidemia prevalence was comparable (AFLD 60.0% (18/30), MASLD 60.0% (18/30), p<0.001 versus controls). Patients with AFLD reported a mean alcohol consumption of 65.7±22.1 g/day.

Conclusion: MASLD manifests earlier with dense metabolic clustering, while AFLD presents later with significant alcohol exposure. These distinct phenotypes suggest divergent pathogenic trajectories requiring tailored screening and management. The high metabolic burden in AFLD underscores overlapping etiologies and potential synergistic hepatotoxicity.

## Introduction

Steatotic liver disease encompasses a spectrum of conditions characterized by excessive hepatocellular lipid accumulation, historically dichotomized into alcoholic fatty liver disease (AFLD) and non‑alcoholic fatty liver disease (NAFLD). The recent paradigm shift to metabolic dysfunction‑associated steatotic liver disease (MASLD) nomenclature better reflects the metabolic underpinnings of the non‑alcoholic variant [1]. Globally, MASLD prevalence approaches 25‑30%, paralleling the obesity and metabolic syndrome pandemic, while AFLD remains a significant contributor to cirrhosis and liver‑related mortality [2-4].

India faces a dual burden: traditional alcohol‑related liver disease coexists with rapidly escalating metabolic liver disease, with overall fatty liver prevalence estimated at 15‑32% [5]. Unique genetic, dietary, and socioeconomic factors may modulate disease expression differently than in Western populations [6]. Despite shared histopathological features, AFLD and MASLD diverge in etiopathogenesis: direct alcohol toxicity versus insulin resistance‑mediated metabolic dysregulation [7,8]. Comparative epidemiological studies from South Asia, particularly India, remain sparse. Existing literature largely examines these entities separately, with limited direct comparisons of demographic and metabolic profiles within the same population [9,10]. Understanding phenotype differences is crucial for developing population‑specific screening algorithms, prevention strategies, and resource allocation [11]. Accordingly, the present study was conducted to characterize and compare the demographic, anthropometric, and metabolic profiles of patients with AFLD and MASLD in a Western Indian population.

## Materials and methods

Study design and ethical considerations

This analytical cross-sectional study was conducted from June 2023 to December 2024. This study was approved by the Institutional Ethics Committee of Krishna Vishwa Vidyapeeth (Deemed to be University), Karad, India (approval no. KVV/IEC/03/2024). All participants provided written informed consent. The study adhered to the STROBE (Strengthening the Reporting of Observational Studies in Epidemiology) guidelines for observational research.

Study population

Inclusion Criteria

Inclusion criteria included patients aged 18-80 years. The AFLD group consisted of individuals with alcohol consumption of 30 g/day or more for men or 20 g/day or more for women for five years or longer, along with hepatic steatosis confirmed on ultrasonography. The MASLD group included individuals with hepatic steatosis on ultrasonography, no alcohol consumption, and at least one metabolic risk factor. The control group comprised individuals with no hepatic steatosis, no alcohol consumption, and no components of metabolic syndrome.

Exclusion Criteria

Exclusion criteria included other chronic liver diseases (viral hepatitis B or C, autoimmune hepatitis, Wilson's disease, hemochromatosis); acute hepatic decompensation or hepatocellular carcinoma; malignancy or active inflammatory conditions; pregnancy or lactation; refusal to provide informed consent; and use of lipid-lowering medications within three months.

Operational Definitions

Hepatic steatosis was diagnosed using established ultrasonographic criteria, including liver-kidney contrast discrepancy, parenchymal brightness, deep attenuation, and vessel blurring [12]. Significant alcohol consumption was defined as ≥30 g/day for men or ≥20 g/day for women for a duration of five years or longer [13]. Diabetes mellitus was defined as fasting plasma glucose ≥126 mg/dL, HbA1c ≥6.5%, or documented antidiabetic treatment [14]. Hypertension was defined as blood pressure ≥130/85 mmHg or use of antihypertensive medication [15]. Dyslipidemia was defined as any abnormality in lipid parameters or current lipid-lowering treatment. Obesity was defined using an Asian-specific cutoff of body mass index (BMI) ≥25 kg/m² [16].

Data Collection Procedures

Data were collected using a structured, pre‑tested proforma that captured multiple domains of participant information. Demographic data included age, sex, education, occupation, and socioeconomic status as assessed by the Modified Kuppuswamy scale. Clinical measurements comprised height, weight, BMI, waist circumference, and blood pressure recorded as the average of two consecutive readings. Medical history was documented through a combination of hospital medical records and validated questionnaires. Alcohol consumption was quantified using the timeline follow‑back method and subsequently converted to grams per day using standard conversion factors [17]. Finally, ultrasonography was performed by experienced radiologists who were blinded to the clinical data, using Mindray DC‑70 ultrasound systems to assess the presence and grade of hepatic steatosis.

Participant Flow

**Figure 1:**
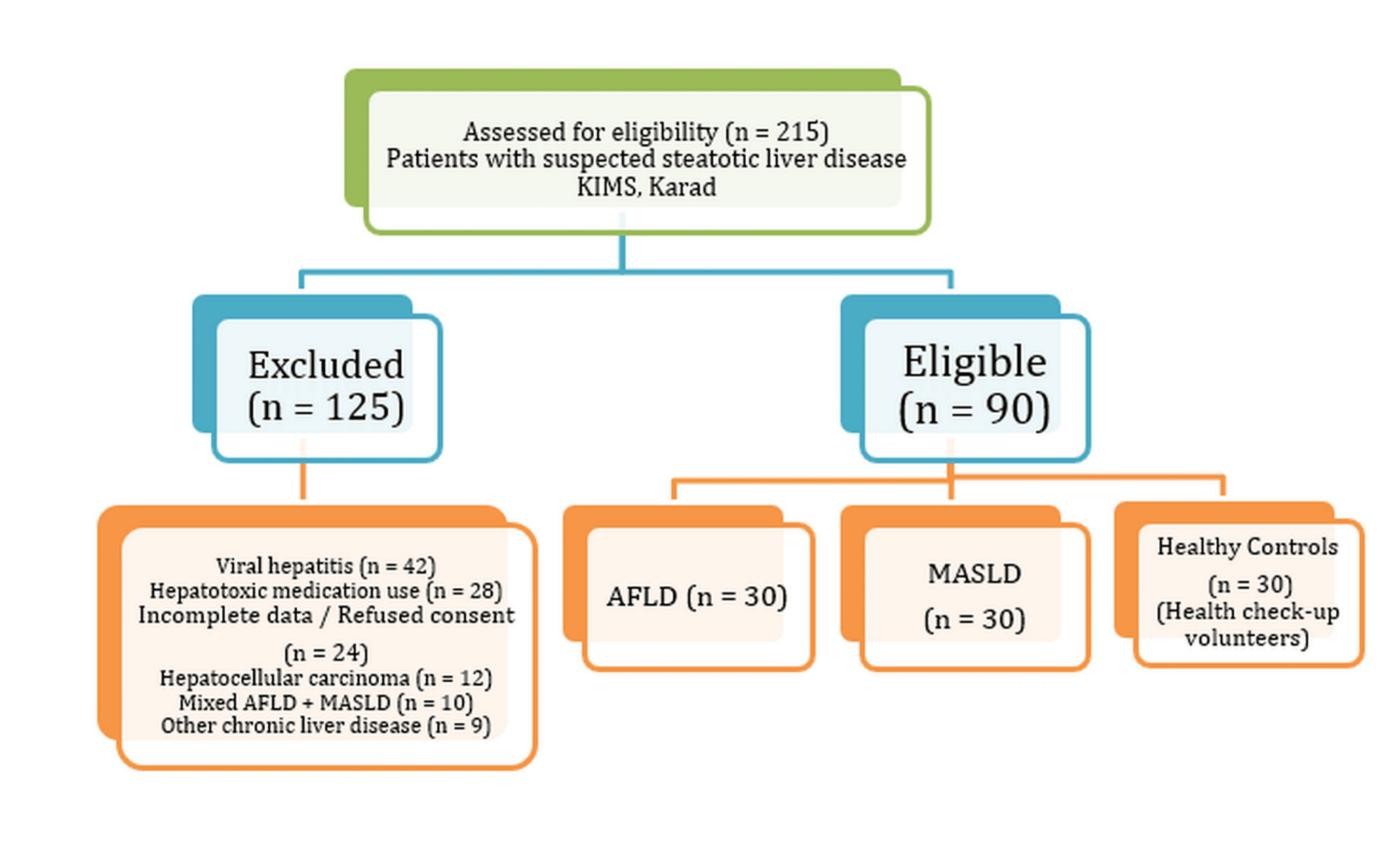
Participant Flowchart: Screening, Exclusions, and Final Allocation Participant screening and enrollment flowchart: Screened (n = 215) → Excluded (n = 125) for viral hepatitis (42), hepatotoxic drugs (28), incomplete data/refusal (24), hepatocellular carcinoma (12), mixed etiology (10), other chronic liver disease (9) → Final analysis (n = 90) divided into AFLD (30), MASLD (30), and healthy controls (30). AFLD: alcoholic fatty liver disease; MASLD: metabolic-associated steatotic liver disease No artificial intelligence (AI) tool was used to create the image.

All consecutive adult patients attending the hospital between June 2023 and December 2024 were screened for eligibility. A total of 215 individuals underwent initial screening based on routine clinical evaluation and ultrasonography reports. Patients meeting the preliminary inclusion criteria (age 18-80 years, presence or absence of hepatic steatosis on ultrasound) were approached by a study physician and provided written information about the study. Those providing written informed consent underwent detailed clinical, anthropometric, and laboratory assessment as described below. The final analytical sample consisted of 90 participants who met all inclusion criteria and had no exclusion criteria. Figure 1 provides a detailed participant flowchart.

Statistical analysis

Data were analyzed using IBM SPSS Statistics version 25.0 (released 2017; IBM Corp., Armonk, New York, United States). Continuous variables were tested for normality using the Shapiro‑Wilk test. Normally distributed variables are expressed as mean ± standard deviation (SD) and compared using one‑way analysis of variance (ANOVA) with post‑hoc Bonferroni correction. Non‑normal variables are expressed as median (interquartile range (IQR)) and compared using the Kruskal‑Wallis test. Categorical variables are expressed as frequencies (percentages) and compared using chi‑square or Fisher’s exact test. Binary logistic regression identified independent predictors of disease phenotype. All tests were two‑tailed with α=0.05.

## Results

Participant flow and baseline characteristics 

Out of a total of 215 screened individuals, 90 met the inclusion criteria (41.8% eligibility rate). Primary reasons for exclusion were viral hepatitis (n=42), hepatotoxic medication use (n=28), incomplete data or refusal of consent (n=24), hepatocellular carcinoma (n=12), mixed alcohol-associated and metabolic dysfunction-associated steatotic liver disease (n=10), and other chronic liver diseases (n=9). Table 1 presents comprehensive baseline characteristics.

**Table 1:** Comprehensive Baseline Characteristics of Study Participants Data are presented as mean ± standard deviation (SD) for continuous variables and as number (n) with percentage (%) for categorical variables. For continuous variables, one-way ANOVA was used with F-statistics reported. For categorical variables, the Chi-square (χ²) test was used. Post-hoc comparisons were performed using Tukey's honest significant difference (HSD) test for continuous variables, and pairwise Chi-square tests with Bonferroni correction for categorical variables. Only statistically significant pairwise differences (p<0.05) are shown. Inequality signs (e.g., A > M, C) indicate which groups differ, with the corresponding p-value in parentheses. The asterisk (*) indicates statistically significant pairwise differences between groups (p < 0.05) identified during post-hoc comparisons. AFLD: alcoholic fatty liver disease; MASLD: metabolic dysfunction-associated steatotic liver disease; Control: healthy controls; BMI: body mass index; MetS: metabolic syndrome; A: AFLD group; M: MASLD group; C: Control group

Characteristic	AFLD (n=30)	MASLD (n=30)	Control (n=30)	Test statistic	p-value	Post-hoc comparisons*
Demographic						
Age (years)	52.5 ± 10.2	45.1 ± 9.8	48.3 ± 8.5	F = 3.52	0.032	A > M (p=0.028)
Anthropometric						
BMI (kg/m²)	31.2 ± 3.5	32.8 ± 4.1	24.5 ± 2.8	F = 42.6	<0.001	A, M > C (p<0.001)
Waist circumference (cm)	98.5 ± 8.2	102.3 ± 9.1	86.4 ± 6.8	F = 28.4	<0.001	A, M > C (p<0.001)
Lifestyle						
Alcohol (g/day)	65.7 ± 22.1	2.1 ± 3.5	1.8 ± 2.9	F = 98.3	<0.001	A > M, C (p<0.001)
Smoking, n (%)	17 (56.7)	8 (26.7)	6 (20.0)	χ² = 11.9	<0.001	A > M, C (p<0.001)
Metabolic comorbidities						
Diabetes, n (%)	12 (40.0)	14 (46.7)	4 (13.3)	χ² = 12.4	0.002	A, M > C (p<0.01)
Hypertension, n (%)	15 (50.0)	16 (53.3)	8 (26.7)	χ² = 10.8	0.005	A, M > C (p<0.01)
Dyslipidemia, n (%)	18 (60.0)	18 (60.0)	6 (20.0)	χ² = 16.2	<0.001	A, M > C (p<0.001)
Metabolic syndrome						
Full MetS, n (%)	11 (36.7)	13 (43.3)	2 (6.7)	χ² = 14.5	<0.001	A, M > C (p<0.001)
Components, mean ± SD	2.8 ± 1.1	3.2 ± 0.9	0.8 ± 0.7	F = 22.7	<0.001	A, M > C (p<0.001)

Detailed analysis of metabolic parameters

Age distribution analysis revealed that patients with MASLD clustered in the 35-55 year range (63.3%, 19/30), while those with AFLD were distributed more evenly across 40-65 years (Figure 1). The 10‑year age difference between groups was statistically significant (p=0.032). Both disease groups exhibited central obesity patterns. Waist circumference exceeded Asian cutoffs (≥90 cm for men, ≥80 cm for women) in 90% (27/30) of patients with MASLD and 87% (26/30) of patients with AFLD. Waist‑to‑height ratio (mean: AFLD 0.58, MASLD 0.61) indicated high cardiometabolic risk in both groups. Metabolic syndrome prevalence followed a gradient: MASLD (43.3%, 13/30) > AFLD (36.7%, 11/30) > controls (6.7%, 2/30). Patients with MASLD exhibited tighter clustering, with 60% (18/30) having ≥ three components versus 47% (14/30) in AFLD. Hypertension and dyslipidemia showed the strongest association with the MASLD phenotype.

Regression analysis

Binary logistic regression identified independent predictors:

For MASLD versus controls: younger age (OR: 0.92, 95% CI: 0.87-0.97), diabetes (OR: 5.8, 95% CI: 2.1-16.2), central obesity (OR: 4.3, 95% CI: 1.9-9.8)

For AFLD versus controls: older age (OR: 1.08, 95% CI: 1.02-1.14), alcohol dose (OR: 1.12, 95% CI: 1.06-1.19 per 10 g), smoking (OR: 3.2, 95% CI: 1.3-7.9)

For MASLD versus AFLD: younger age (OR: 0.91, 95% CI: 0.86-0.96), diabetes (OR: 2.1, 95% CI: 1.1-4.2), absence of smoking (OR: 0.3, 95% CI: 0.1-0.7)

Notably, despite stratification efforts, a significant age difference persisted between AFLD and MASLD groups (52.5 vs. 45.1 years; p=0.032), which was adjusted for in multivariable regression.

## Discussion

This study, conducted with 90 participants (30 per group), reveals distinct phenotypic patterns between AFLD and MASLD in Western India. MASLD’s earlier presentation (mean age: 45 years) aligns with metabolic dysfunction often manifesting in early middle age, while AFLD’s later presentation (53 years) reflects cumulative alcohol toxicity [18,19]. The 8‑year age gap exceeds reports from Western cohorts (typically 3‑5 years), suggesting population‑specific dynamics [20].

The high metabolic burden in AFLD (40% (12/30) diabetes, 50% (15/30) hypertension) challenges traditional dichotomization. Recent evidence suggests alcohol and metabolic dysfunction may synergistically accelerate liver injury through shared pathways: oxidative stress, gut dysbiosis, and adipose tissue inflammation [21,22]. Our findings support an integrated “metabolic‑alcoholic” spectrum rather than distinct entities.

Comparative analysis with global literature

Our MASLD cohort showed higher metabolic syndrome prevalence (43.3%, 13/30) than reported in some Indian studies (25‑35%) [23], possibly reflecting selection bias toward symptomatic patients. The diabetes prevalence (46.7%, 14/30) aligns with global MASLD estimates (40‑50%) [24]. Patients with AFLD exhibited higher metabolic comorbidities than typically reported in Western literature (20‑30% diabetes) [25], suggesting potential ethnic differences in alcohol‑metabolism interactions. Despite the smaller sample size (n=30 per group), the effect sizes remained large enough to detect statistically significant differences, indicating robust phenotypic separation.

Pathophysiological implications

The phenotypic differences may reflect distinct pathogenic sequences:

MASLD: primarily “outside‑in” pathology where systemic metabolic dysfunction drives hepatic steatosis via insulin resistance, adipokine imbalance, and lipotoxicity [26].

AFLD: primarily “inside‑out” pathology where direct hepatocyte alcohol metabolism generates toxic metabolites (acetaldehyde, reactive oxygen species), with secondary metabolic consequences [27]. The overlap suggests convergent pathways, possibly mediated by gut‑liver axis alterations, mitochondrial dysfunction, and endoplasmic reticulum stress [28].

Clinical and public health implications

Screening strategies: MASLD presents at a younger mean age (45.1 years) compared to AFLD (52.5 years), suggesting that if age-based screening were to be considered, further prospective studies would be needed to determine optimal cutoffs, while AFLD screening should target older individuals with alcohol use patterns. With 30 patients per group, the age difference remained significant, supporting age‑based screening algorithms.

The high metabolic comorbidity burden in both groups (diabetes 40-47%, hypertension 50-53%) indicates that metabolic assessment is relevant regardless of etiology. Whether integrated management improves outcomes requires interventional studies.

Prevention: Public health interventions should address both alcohol consumption and metabolic health simultaneously.

Study limitations (with n=30 per group)

The sample size may limit generalizability and statistical power for subgroup analyses. However, key differences remained significant, suggesting the observed effects are clinically meaningful. The lack of formal age matching between disease groups represents a potential source of residual confounding, although regression analysis was used to mitigate this. Future larger cohort studies are warranted to validate these findings.

## Conclusions

This cross-sectional study of 90 participants from Western India demonstrates that AFLD and MASLD exhibit distinct demographic and metabolic profiles despite both being associated with hepatic steatosis. Patients with MASLD presented at a significantly younger mean age (45.1 ± 9.8 years) compared to those with AFLD (52.5 ± 10.2 years; p=0.032). Both disease groups showed significantly higher BMI, waist circumference, and prevalence of diabetes, hypertension, and dyslipidemia compared to healthy controls (p<0.001 for each). Metabolic syndrome prevalence was elevated in both groups (MASLD 43.3%, AFLD 36.7%) compared to controls (6.7%; p<0.001). Notably, the AFLD group demonstrated substantial metabolic comorbidity despite the absence of metabolic syndrome as an inclusion criterion, with 40% diabetes, 50% hypertension, and 60% dyslipidemia. Binary logistic regression identified younger age and diabetes as independent predictors of MASLD versus AFLD, while alcohol dose and smoking predicted AFLD. These findings indicate that while MASLD presents as an earlier-onset metabolic clustering phenotype, AFLD in this Western Indian population carries a significant concomitant metabolic burden that challenges the traditional dichotomous classification of steatotic liver disease by etiology alone. Further prospective studies with larger sample sizes are warranted to explore the potential overlapping pathogenic mechanisms suggested by these phenotypic observations.
